# Advances in targeted liquid chromatography‐tandem mass spectrometry methods for endocannabinoid and N‐acylethanolamine quantification in biological matrices: A systematic review

**DOI:** 10.1002/mas.21897

**Published:** 2024-07-03

**Authors:** Khalisa Amir Hamzah, Natalie Turner, David Nichols, Luke J. Ney

**Affiliations:** ^1^ School of Psychology and Counselling, Department of Health Queensland University of Technology Kelvin Grove Queensland Australia; ^2^ The Centre for Children's Health Research Queensland University of Technology Kelvin Grove Queensland Australia; ^3^ Central Science Laboratory, Science and Engineering University of Tasmania Hobart Tasmania Australia

**Keywords:** anandamide, endocannabinoids, LC‐MS, liquid chromatography, mass spectrometry

## Abstract

Liquid chromatography paired with tandem mass spectrometry (LC‐MS/MS) is the gold standard in measurement of endocannabinoid concentrations in biomatrices. We conducted a systematic review of literature to identify advances in targeted LC‐MS/MS methods in the period 2017–2024. We found that LC‐MS/MS methods for endocannabinoid quantification are relatively consistent both across time and across biomatrices. Recent advances have primarily been in three areas: (1) sample preparation techniques, specific to the chosen biomatrix; (2) the range of biomatrices tested, recently favoring blood matrices; and (3) the breadth of endocannabinoid and endocannabinoid‐like analytes incorporated into assays. This review provides a summary of the recent literature and a guide for researchers looking to establish the best methods for quantifying endocannabinoids in a range of biomatrices.

## 1 INTRODUCTION

In the late 20th century, the discovery of a receptor class with specific affinity for cannabinoids sparked investigation into the endogenous cannabinoid system (Devane, 1988, 1992; Howlett, 2002). The endogenous cannabinoid (endocannabinoid) system is a lipid signalling system consisting of fatty acid neurotransmitters known as endocannabinoids, enzymes that participate in the endocannabinoid biosynthesis pathway, and G protein‐coupled receptors that are specifically responsive to endocannabinoid neurotransmitters (Di Marzo, 1994; Howlett, 2002; Kogan & Mechoulam, 2006; Mechoulam & Parker, 2013; Munro et al., 1993; Suguira, 1995).

Two endocannabinoid neurotransmitters that have affinity for cannabinoid receptors have been discovered to date: arachidonoyl ethanolamide (AEA) and 2‐arachidonoyl glycerol (2‐AG) (Devane, 1992; Suguira, 1995). While AEA and 2‐AG are the only endocannabinoids known to directly interface with the cannabinoid receptors, additional endocannabinoid‐like N‐acylethanolamines neurotransmitters also include oleoylethanolamide (OEA), palmitoylethanolamide (PEA), stearoylethanolamide (SEA), and linoleoylethanolamide (LEA) (Kogan & Mechoulam, 2006; Mechoulam & Parker, 2013). Endocannabinoids are highly hydrophobic in nature, with low aqueous mobility and solubility, therefore tending to absorb onto plastic and glassware (Dincel, Rosales‐Solano, et al., 2023; Dincel, Zeinali, et al., 2023). Steps are usually taken during sample preparation to stabilise the hydrophobic compounds, such as solid phase extraction (SPE), liquid–liquid extraction, or protein precipitation.

The endocannabinoid system is both a profuse and extremely integral system in both the central and peripheral nervous systems (Ligresti et al., 2016). Understanding of human health across many medical fields has consequently been affected by the discovery of the endocannabinoid system, including cancer, immune functioning, inflammation, neurological disorders, cardiovascular health, and many more (Amir Hamzah, 2023; Di Marzo & Petrosino, 2007). Unexpectedly, the endocannabinoid system was also discovered to be involved in the fundamental processes of neural plasticity (Kano, 2009; Ohno‐Shosaku et al., 2001) and has since been hypothesised to play a role in most psychiatric disorders (Di Marzo et al., 2015; Mechoulam & Parker, 2013; Ney, 2022; Ney, Crombie, et al., 2023; Scherma, 2020). For these reasons, the endocannabinoid system is of increasing important to medical research.

Techniques for endocannabinoid quantification have been successfully applied to human blood, plasma, serum, cerebral spinal fluid, brain tissue, breast milk, meconium, urine, saliva, hair, nails, and reproductive fluids (Battista, 2014; Marchioni, 2018). Quantification can be achieved using hyphenated mass spectrometry techniques, and previous research has shown that use of liquid chromatography–mass spectrometry (LC‐MS) is more efficient than gas chromatography‐MS, due to unstable derivatization of 2‐AG (Zoerner, 2009, 2011). Microdialysis is also not a straightforward analytical option for endocannabinoid quantification, since this method relies on an aqueous environment that is not compatible with endocannabinoids, which are water‐insoluble fatty acids (Buczynski & Parsons, 2010; Marchioni, 2018; Wiskerke, 2012; Zestos & Kennedy, 2017).

Recent reviews have described available LC‐MS assays that can be used to quantify endocannabinoids in human biomatrices (Battista, 2014; Marchioni, 2018) and have comprehensively evaluated the technical specifications underlying successful analysis of these lipids at most levels of the processing pipeline (Kratz et al., 2021; Thieme, 2020; Zoerner, 2011). These issues will not be re‐reviewed here. However, techniques for endocannabinoid quantitation have improved considerably over the past decade. The current systematic review therefore provides an exhaustive and up‐to‐date review of the most recent LC‐MS analytical approaches of endocannabinoid analysis in human biomatrices. This article will benefit researchers and laboratories seeking to adopt endocannabinoid analyses by providing current information on the best available methods for a range of biomatrices.

## 2 METHOD

### 2.1 Search strategy

A systematic literature search of Web of Science and Scopus using the search terms “[Title/Abstract} Endocannabinoid*” OR “[Title/Abstract] endogenous cannabinoid*” AND “[Title/Abstract] LC‐MS*” OR “[Title/Abstract] spectrometry” was conducted. The search period was from 2017 to 2024 and was conducted on the 20th of November 2023. All articles were compiled and screened using Rayyan software (Ouzzani, 2016).

### 2.2 Study eligibility

Articles were included in this review if they (1) used novel liquid chromatography paired with tandem mass spectrometry methods, (2) analysed endocannabinoids in human biomatrices, (3) used targeted multiple reaction monitoring methods, (4) were conducted between January 2017 and January 2024, (5) were journal articles, book chapters or theses, and (6) were published in English. Articles were excluded in this review if they (1) did not have English full text, or (2) were review articles.

### 2.3 Data extraction

Title and abstract screening of the initial 788 articles was performed by two independent raters (KAM and LN). All articles were screened by both raters separately based on the inclusion and exclusion criteria. Articles that appeared to meet these criteria were subsequently screened as full texts. In total, 909 articles were identified across the two databases, and, of these, 121 duplicates were removed, leaving 788 articles. Of the 788 articles screened during Title/Abstract screening, 122 articles were deemed eligible for full text screening. Of these, 53 articles were included, and 6 additional articles were found through manual searches (e.g., through reference list and Google Scholar searches).

## 3 RESULTS AND DISCUSSION

### 3.1 Blood samples (including whole blood, plasma, and serum)

Blood is one of the most accessible forms of biological matrices for the quantification of endocannabinoids. As first reported by Giuffrida, Rodríguez de Fonseca (Giuffrida et al., 2000a), AEA, PEA, and OEA were quantified on a C18 column using simple mobile phases, with water as phase A and methanol as phase B. As more assays have been developed, a C18 analytical column has been largely retained, as shown in Table 1. Although sub‐2µm particle size C18 columns are not necessary for measurement of endocannabinoids in blood samples, our review shows that this column parameter is most frequently used. While water as mobile phase A has also remained consistent, small percentages of formic acid, acetic acid, or ammonium acetate have been incorporated as mobile phase modifiers. Likewise, the addition of small percentages of formic acid to methanol in mobile phase B have been reported, though to a lesser extent. Instead, more recent assays have used an acetonitrile base for mobile phase B, as shown in Table 1. Karu (2022) and Ortiz‐Alvarez (2022) report the addition of a mobile phase C, both using isopropanol containing 0.1% acetic acid, though typically methods retain a standard binary pump LC operation. In the following section, we describe some of the most innovative new approaches in detail, with other more typical approaches primarily described in Table 1.

**Table 1:** Analytical methods and parameters of recent methods for quantifying endocannabinoids in blood using LC‐MS.

Study	Analyte(s)	Extraction	Injection	Column	Flow Rate	Mobile Phase A	Mobile Phase B	Ionisation	LC System	MS System	LLOQ
Acquaro Junior et al. (2019)	AEA, 2‐AG	2 h DI‐SPME (1500 rpm orbital agitation)	10 μL	C18 (1.6 μm, 100 mm × 2.1 mm)	0.3 mL/min	0.1% of CH₃COOH	MeCN/C_3_H_8_O (90:10, v/v)	ESI in positive ion mode	Thermo Dionex UltiMate 3000	TSQ Quantiva	1 ng/mL
Archambault (2021)	13‐HODE, 1‐LG, LA	Homogenisation and centrifugation with 500 µL MeOH containing internal standards and acidified with 0.575% CH₃COOH and CHCI_3._ Organic phase is dried and reconstituted with 25 µL each of the mobile phases	40 µL	C8 (2.6 μm, 150 mm × 2.1 mm)	0.4 mL/min	H₂O:1 nM NH₄OH: 0.05% CH_3_COOH	MeCN:H₂O (95:5) containing 1 mM NH_₄_OH and 0.05% CH_3_COOH	ESI in positive ion and negative ion mode	‐	Shimadzu 8050	25 Fmol
Battista et al. (2023)	AEA and 2‐AG	Sonication and centrifugation, then µSPE	‐	C18 (1.7 μm, 100 mm × 2.1 mm)	0.3 mL/min	H₂O	2.5 mM FA in MeOH	ESI in positive ion mode	Nexera XR LC20AD	Sciex Qtrap 4500 hybrid	0.032–2.57 ng/mL
Bilgin and Shevchenko (2017)	1‐AG, 2‐AG, NAGly, NASer	Liquid‐liquid extraction	40 µL	C18 (5 μm, 150 mm × 0.5 mm)	20 μL/min	0.1% FA in H₂O	MeCN: C_3_H_8_O:FA (9:1:0.1)	ESI in positive ion mode	Agilent micro‐LC 110	Thermo Fisher triple quadrupole	‐
Couttas et al. (2023)	AEA, OEA, PEA, 2‐AG, Δ9‐THC	Evaporation with 2:1 CHCI_3_/MeOH	10 µL	C18 (4 μm, 150 mm × 2 mm)	0.5 mL/min	0.1% FA in H₂O	MeOH	ESI in positive ion mode	Agilent 1200 HPLC	Sciex API 5000	0.012–0.24 pmol/mL
Dong (2019)	AEA, 2‐AG, 1‐AG	Homogenisation and centrifugation with 9:1 C₆H₅CH₃ and MTBE. Organic phase is reconstituted with 100 µL MeCN	5 µL	C18 (1.7 μm, 100 mm × 2.1 mm)	0.3 mL/min	0.1% FA in H₂O	MeCN	ESI in positive ion mode	Thermo Dionex UltiMate 3000 UHPLC	Thermo Fisher triple quadrupole	0.05 ng/mL
Gurke (2019)	AEA, OEA, PEA, 1‐AG, 2‐AG	Homogenisation and centrifugation with 9:1 200 µL C_4_H_8_O_2_:C₆H₁₄. Organic phase is evaporated and reconstituted with 50 µL MeCN	‐	C18 (1.7 μm, 100 mm × 2.1 mm)	‐	0.0025% FA in H₂O	0.0025% FA in MeCN	ESI in positive ion mode	Agilent 1290 Infinity LC	Sciex QTRAP 6500+	0.197–1.574 ng/mL
Karu (2022)	AA, AG, LG, LA	Liquid‐liquid extraction using 1:1 C₄H₉OH:MTBE	10 µL	C18 (1.7 μm, 50 mm × 2.1 mm)	0.7 mL/min	H₂O and 0.1% CH_3_COOH	MeCN:MeoH (90:10) containing 0.1% CH_3_COOH	ESI in positive ion and negative ion mode	Shimadzu Nexera X2 UHPLC	Sciex QTRAP 6500	‐
Karhson (2018)	AEA	Lipid extraction with a modified salt‐assisted liquid‐liquid extraction	‐	C18 (1.7 μm, 150 mm × 2.1 mm)	‐	0.1% FA in H₂O	0.1% FA in MeCN	ESI in positive ion mode	Accela 1250 HPLC	TSQ Vantage	50 fg
Luque‐Córdoba (2018)	SEA, DHEA, DEA, 2‐AG, AEA, NAGly, OEA, PEA	SPE, eluting wih 70% MeCN in H₂O	5 µl	C18 (2.6 μm, 100 mm × 5 mm)	0.8 mL/min	0.1% FA in H₂O	0.1% FA in MeCN	ESI in positive ion mode	Agilent 1200	Agilent 6410	1–150 pg/mL
Marchioni (2017)	AEA and 2‐AG	Liquid‐liquid extraction using 1 mL C₆H₅CH₃	10 μL	C18 (1.7 μm, 100 mm × 2.1 mm)	0.4 mL/min	0.5% FA in H₂O	0.5% FA in MeCN	ESI in positive ion mode	Waters ACQUITY UPLC H‐Class	Xevo® TQ‐D	0.1 ng/mL for AEA 0.04 ng/mL for 2‐AG
Ortiz‐Alvarez (2022)	AEA, 2‐AG, AA, DHEA, LEA, PEA, OEA, SEA, 2‐LG, 2‐OG	Liquid‐liquid extraction using 1 mL 50:50 C₄H₉OH:MTBE	10 μL	C18 (1.7 μm, 50 mm × 2.1 mm)	0.7 mL/min	0.1% CH_3_COOH in H₂O	MeCN/0.1% CH_3_COOH in MeOH (90:10)	ESI in positive ion mode	SCIEX QTRAP® LC‐ESI‐MS/MS System	SCIEX QTRAP® LC‐ESI‐MS/MS System	‐
Pedersen et al. (2021)	AA, OEA, LEA, AEA, DEA, DHEA, 2‐AG, steroids and bile acids	Homogenisation and centrifugation with C_15_H_24_O and EDTA in MeOH, and 5 μM 1‐cyclohexylyredio, 3‐dodecanoic acid, 1‐phenylureido, 3‐hexanoid acid in methanol. 96‐well plate capped with silicone mat and chilled at −20°C for 15 min. Supernatant aliquoted and centrifuged.	5 µl	C18 (1.7 μm, 150 mm × 2.1 mm)	0.5 mL/min	0.1% CH_3_COOH in H₂O	C_3_H_8_O:MeCN(10:90)	ESI in positive ion and negative ion mode	Shimadzu Nexera X2 UPLC	API 6500 QTRAP	0.399 nM for AEA 1.69 nM for 2‐AG ECBs ranging from 0.399–129 nM
Röhrig (2019)	MEA, PEA, SEA, OEA, EEA, VEA, LEA, AEA, DHEA, 2‐PG, 2‐OG, 2‐LG, 1‐LG, 2‐AG, 1‐AG, NAGLy	Homogenisation and centrifugation with 210 µL 20:1 C₆H₅CH₃:C₄H₉OH	24 µL	C18 (1.7 μm, 150 mm × 2.1 mm)	20 µl/min	H₂O: C_3_H_8_O: CH_3_COOH (65:35:0.05)	MeCN with 0.05% CH_3_COOH	ESI in positive ion mode	Dionex UltiMate 3000 RS, Thermo Fisher Scientific	API 4000 QTRAP coupled to a pump for direct infusion (Harvard Apparatus 11plus)	0.032–0.64 ng/mL for ECBs
Sempio (2021)	AEA, 2‐AG, 1‐AG, DEA, LEA, OEA, PEA, SEA	Homogenisation and centrifugation with MeCN and internal standard. Supernatant reconstituted with 0.1% FA in H₂O	400 µL	EC18 (2.7 μm, 50 mm × 4.6 mm)	0.8 mL/min	0.1% FA in H₂O	0.1% FA in MeCN	APCI in positive ion mode	Agilent 1200 Series LC/LC with online‐trapping column	Sciex API5500	0.05–2.5 ng/mL
Souza et al. (2019)	AEA and 2‐AG	Protein precipitation with MeCN. Supernatant reconstituted with 90:10 5 mM C₂H₇NO₂ and MeCN	400 μL	C18 (1.7 μm, 100 mm × 2.1 mm)	0.1 mL/min	H₂O	0.5% FA in H₂O	ESI in positive ion mode	Waters ACQUITY UPLC H‐Class	Xevo® TQ‐D	0.10 ng/mL for AEA 0.05 ng/mL for 2‐AG
Tagliamonte et al. (2020)	AEA, 2‐AG, OEA, LEA, PEA, SEA, LA, AA	Homogenisation and centrifugation with 1.5 mL 2:1 CHCl_3_:CH_3_OH. Supernatant aliquoted twice, then CHCl_3_ layer evaporated and reconstituted with 100 μL MeCN:C_3_H_8_O:H₂O (60:35:5)	10 μL	C18 (2.6 μm, 100 mm × 2.1 mm)	0.2 mL/min	H₂O:MeCN (40:60) with 5 mM NH_4_HCO_2_ and 0.1% FA)	C_3_H_8_O:MeCN (90:10) with 5 mM NH_4_HCO_2_ and 0.1% FA)	ESI in positive ion and negative ion mode	Accela U‐HPLC	Exactive Orbitrap MS	0.25–2.5 ng/mL
Villate (2023)	1‐AG, 2‐AG, AEA, PEA, LEA, OEA, DEA	Homogenisation and centrifugation with 1 mL MTBE. Organic phase is evaporated and reconstituted with 200 μL MeCN	10 μL	C18 (2.6 μm, 150 mm × 3 mm)	0.3 mL/min	0.1% FA in H₂O	0.1% FA in MeOH	ESI in positive ion mode	1290 Infinity II liquid chromatography	6430 Triple Quad (with an Edwards 28 E2M28 vacuum pump and a RMSN‐2 big universal trap for nitrogen)	‐

Abbreviations: 2‐OG, 2‐oleoylglycerol; 2‐PG, 2‐palmitoyl glycerol; 13‐HODE‐EA, 13‐hydroxy‐9Z,11E‐octadecadienoyl‐N‐ethanolamine; AA, arachidonic acid; AEA, arachidonoyl ethanolamide; AG, arachidonoyl glycerol; APCI, atmospheric pressure chemical ionization; CHCI_3_, chloroform; C₂H₇NO₂, ammonium acetate; C_3_H_8_O, isopropanol; CH_3_COOH, acetic acid; C_4_H_8_O_2_, ethyl acetate; C₄H₉OH, butanol; C₆H₁₄, hexane; C₆H₅CH₃, toluene; C_15_H_24_O, butylated hydroxytoluene; DEA, docosatetraenoyl ethanolamide; DHEA, docosahexaenoyl ethanolamide; DI‐SPME, direct immersion solid‐phase microextraction; ECB, endocannabinoid; EDTA, ethylenediaminetetraacetic acid; EEA, elaidic acid ethanolamide; ESI, electrospray ionisation; FA, formic acid; H₂O, water; LA, linoleic acid; LEA, linoleylethanolamine; LG, linoleoylglycerol; MEA, methanandamide; MeCN, acetonitrile; MeOH, methanol; MTBE, methyl tert‐butyl ether; NAGly, N‐arachidonoyl glycine; NAPE, N‐acylphosphatidyl ethanolamine; NASer, N‐arachidonoyl serine; NH₄OH, ammonium hydroxide; NH_4_HCO_2_, ammonium formate; OEA, oleoylethanolamide; PEA, palmitoylethanolamide; RPM, revolutions per minute; SEA, stearoylethanolamine; VEA, vaccenic acid ethanolamide; Δ9‐THC, delta‐9‐tetrahydrocannabinol.

Marchioni (2017) designed an assay for the quantification of AEA and 2‐AG in plasma, using the novel method of column‐switching. This included using a restricted access media column for the first dimension of LC, then switching to the C18 column in the second dimension. The restricted access media column allowed for reverse phase partitioning for trace enrichment of the endocannabinoids, while the C18 standard column chromatographically separated the analytes. This resulted in coefficients of variation lower than 8%, and Lower Limits of Quantification (LLOQs) of 0.1 and 0.04 ng/mL for AEA and 2‐AG, respectively. However, the chromatographic resolution was suboptimal in this study, with inability to separate 1‐AG from 2‐AG due to 1‐min wide peaks.

Pedersen et al. (2021) quantified endocannabinoids along with bile acids, steroids, and fatty acids from serum and plasma using a novel 96‐well plate format. This format relied on protein precipitation and filtration, and increased the efficiency of the sample preparation method, especially for large lists of targets. The 96‐well plates were capped with a slit‐top silicone mat which allowed analysis of sample extracts directly from plate wells in the Shimadzu Nexera X2 UPLC coupled with the API 6500 QTRAP. More recently, the silicone mats have been upgraded to thermally sealed polypropylene‐backed foil. This novel format for preparing the sample extracts for analysis allowed for LLOQs of 0.399 nM for AEA, 1.69 nM for 2‐AG, and other related N‐acylethanolamines ranging from 0.399 to 129 nM. While lower LLOQs have since been reported (e.g., an LLOQ of 0.032 ng/mL for AEA and 2‐AG; Battista et al., 2023), and this method is not as sensitive when compared to targeted assays such as Marchioni (2017), this format allows for a larger investigation of metabolic cross‐interactions with multiple mediators.

For most assays, injection volume ranged between 5 and 40 µL, though larger targeted lipidomic studies such as Sempio (2021) report 400 µL. This method quantified AEA, 1‐AG, 2‐AG, OEA, PEA, and lesser related endocannabinoids and neuropolypeptides. Sempio (2021) reported LLOQs within the range of 0.05–2.5 ng/mL, which has a slightly high upper range compared to other recent assays such as Marchioni (2017), and as shown in Table 1, though is not unexpected due to the lipidomic approach and shows that endocannabinoids can still be quantified at biologically relevant ranges using lipidomics. Its sample preparation was also largely accessible, in the form of simple protein precipitation from K2‐EDTA plasma and injected into an online extraction column.

The majority of assays (72%) opted for electrospray ionisation (ESI) in positive ion mode only. Some recent studies (22%) also included ESI in negative ion mode, though these included larger analyte lists with lesser related neuropolypeptides (Archambault, 2021; Pedersen et al., 2021). Previous studies suggest that most endocannabinoids can be sufficiently analysed with ESI in positive ion mode only with appropriate LLOQs, as shown in Table 1.

All assays immediately stored fresh blood samples on ice. Due to the high abundance of soluble and secreted factors found in plasma that interfere with targeted lipidomic assays, a number of sample preparation strategies have been employed to improve the extraction of endocannabinoids from blood. Just over half (56%) of recent assays included in this review opted to prepare and extract blood samples using some form of protein precipitation with homogenisation of the internal standard and extraction solvents. This was typically followed by centrifugation, evaporation, and reconstitution with acetonitrile or a mobile phase. For example, Dong (2019) homogenised the samples with 20 μL of the internal standard working solution of 10 ng/mL AEA‐d_4_ in acetonitrile and a 1 mL solvent containing 9:1 toluene and methyl tert‐butyl ether, to extract and quantify AEA, 2‐AG, and 1‐AG. In a novel 96‐well format Pedersen et al. (2021) extracted arachidonic acid, OEA, LEA, AEA, docosatetraenoylethanolamide, docosahexaenoylethanolamide, 2‐AG, and other steroids and bile acids in a similar manner. Samples were added to methanol washed polypropylene 1 mL deep well plates containing 10 μL methanolic solutions of 625 nM deuterated surrogates of endocannabinoids and other targets, 5 μL of 0.2 mg/mL of butylated hydroxytoluene and ethylenediaminetetraacetic acid in 1:1 methanol and water, 5 μL of methanol solutions of 5 μM 1‐cyclohexyluredio, 3‐dodecanoic acid, 1‐phenylureido, and 3‐hexanoic acid, and 170 μL 1:1 methanol and acetonitrile. After homogenisation and centrifugation, the supernatant was aliquoted and pipetted into a 0.2 μm polyvinylidene difluoride membrane filter plate placed over 450 μL microtiter plates and capped with thermally sealed polypropylene‐backed foil.

The remaining assays prepared and extracted the plasma samples through liquid‐liquid extraction (28% of included studies) or a form of SPE (17% of included studies). For example, to quantify 1‐AG, 2‐AG, *N*‐acylethanolamines, *N‐*acylglycine, and *N*‐acylserine, Bilgin & Shevchenko (2017) used a form of liquid‐liquid extraction. Five hundred microliters of samples were homogenised with 750 μL 9:1:0.1 ethyl acetate, n‐hexane, and formic acid, along with 12.5 μL of 25 μM PF3845 and 50 μL of the internal standard working solution, which contained d4‐*N*‐acylethanolamine, d8‐*N*‐acylethanolamine, d8‐*N*‐acylglycine, d8‐2‐acylglycerol, d5‐1‐acylglycerol diluted 10 times in acetonitrile. The solutions were incubated on dry ice for 10 min, and the upper organic phase was aliquoted and evaporated to dryness. The dried extract was reconstituted with an 85 μL mixture of water, acetonitrile, isopropanol, and formic acid. For the quantification of AEA and 2‐AG, Battista et al. (2023) homogenised 10 μL of plasma with 90 μL of water and 100 μL of internal standard solution consisting of 200 mM formic acid in ice‐cold methanol with 1 ng/mL AEA and 200 ng/mL 2‐AG. The upper phase was aliquoted in preparation for micro‐solid phase extraction (μSPE). The analytes are eluted with 50 μL of 10 mM formic acid in methanol.

#### 3.1.1 Summary: The best methods for blood samples

Both novel and well‐validated methodologies are presently available for the quantification of endocannabinoids by LC‐MS/MS. Endocannabinoids typically elute at peak organic solvent flow (mobile phase B), which explains why water is typically used for mobile phase A, while an organic mobile phase B increases in concentration over run time. In that sense, an 8–10‐min runtime has been reported to be sufficient for endocannabinoid analysis, along with a 2–5‐min wash period. Additionally, ionisation is typically reported in ESI positive ion mode across all endocannabinoid analytes in blood, in conjunction with a C18 column. Most methods have used 1.7 μm particle size with 100 mm (or 150 or 50 mm) × 2.1 mm dimensions, though our review shows that measurement of endocannabinoids in blood samples using columns with higher than 2 μm particle size is easily achievable. This may be advantageous given the complex nature of blood samples and the possibility of frequent column clogging with many sample injections with 1.7 μm particle size columns.

Our review shows that, regardless of the method used, recent methods are easily able to quantify endocannabinoids using LC‐MS/MS at biologically relevant levels. However, comparison of these methods shows that challenges in the field remain. Specifically, while methods such as Marchioni (2017) are able to quantify endocannabinoids in blood samples, peak resolution was reported to be poor compared to other methods (e.g., Bilgin & Shevchenko, 2017) that are able to demonstrate crisp chromatographic resolution including complete separation of 2‐AG from its isomer 1‐AG. Acquaro Junior (2019) demonstrate that small choices, including choice of analytical column brand, can affect 2‐AG resolution and typically 2‐AG variability is higher than AEA, presumably due to isomerisation to 1‐AG during sample processing. Marchioni (2017), and to some extent data reported in other studies (e.g., Sempio, 2021) indicate that—as with other analytes in blood samples—there are potential issues with variability of measurements both intra‐ and inter‐day for endocannabinoids. Compared with other methods, these issues may be mitigated—for example, Acquaro Junior (2019) report coefficient of variabilities uniformly lower than 15% for AEA and 2‐AG. This suggests that future studies of LC‐MS/MS‐based endocannabinoid analysis in blood samples should involve identification of ways to improve chromatographic resolution as well as reducing variability of measurement (precision).

### 3.2 Brain samples

Analysis of endocannabinoids in brain samples using mass spectrometry has undergone recent innovation. This innovation has mostly been in sample preparation methods, whereas the instrumentation protocols have remained consistent with previous decades, though a notable exception to this trend was a hybrid Orbitrap method that quantified over 70 endocannabinoid and related analytes (Berman, 2020) (see Table 2). Typically, samples are prepared by homogenisation of brain tissue with extraction solvents, which are later cleaned using SPE. For example, Fanti (2023) extracted samples by homogenising brain tissue with working standard solution of 0.2 mM formic acid in methanol to quantify AEA, 2‐AG, PEA, and OEA, as well as conjugated endocannabinoids and/or AEA metabolites N‐arachidonoyl‐glycine, ‐alanine, ‐dopamine, ‐serine, and ‐γ‐aminobutyric acid. After collection of the supernatant, this research group reported using micro‐SPE to purify samples before analysis using a C18 column, aqueous and alcoholic mobile phases with formic acid, and a triple quadrupole mass spectrometer (Fanti, 2023). Rand (2018) use a similar method to quantify AEA epoxide‐ and diol‐derivatives, though rather than using pipette micro‐SPE, used 3 mL Oasis Hydrophilic‐Lipophilic‐Balanced cartridges for SPE clean‐up. Ahmed (2022) also used tissue homogenisation followed by clean‐up using Captiva Enhanced Matrix Removal Lipid cartridges for the quantification of AEA, 2‐AG, OEA, PEA, and vaccenoylethanolamide (VEA) in rat brain samples. Of these studies, the latter shows the lowest limit of detection and the lowest percentage variation, though Rand (2018) did not report LOD or LLOQ, and both Rand (2018), as well as Fanti (2023) reporting comparatively higher matrix effects and lower precision.

**Table 2:** Analytical methods and parameters of recent methods for quantifying endocannabinoids in brain samples using LC‐MS.

Study	Analyte(s)	Clean‐up	Extraction	Injection	Column	Flow Rate	Mobile Phase A	Mobile Phase B	Ionisation	LC System	MS System	LLOQ
Ahmed (2022)	AEA, 2‐AG, VEA, OEA, PEA	SPE with Captiva EMR Lipid cartridges	Homogenised with 500 µL 1% FA in MeCN	1 µL 0.1% FA in MeOH	C18 (1.8 μm, 50 mm × 2.1 mm)	0.3 mL/min	0.01% FA in H_2_O	0.01% FA in MeOH	ESI in positive ion mode	Agilent 1260 series	Agilent 6460 C triple quadrupole	0.05–1 ng/mL
Aslam (2019)	AEA, 2‐AG	‐	SPME^ with MeCN: H_2_O 3:1	10 µL MeCN: H_2_O 3:1	Ethylene hybrid octadecyl column (1.7 μm, 50 mm × 2.1 mm)	0.3 mL/min	10 mM NH_4_CH_3_CO_2_ in H_2_O	10 mM NH_4_CH_3_CO_2_ in MeCN	ESI in positive ion mode	Waters Acquity UPLC	Sciex TripleTOF 6600	<0.05 ng/mL
Berman (2020)	AEA, 2‐AG, OEA, PEA, LEA, SEA#	C8 SPE, filtered at 0.22 µm	Homogenised in MeOH: MeCN: AcOH 50:50: 0.1	10 µL ethanol	C18 (2.7 μm, 150 mm × 2.1 mm)	0.25 mL/min	0.1 AcOH in H_2_O	0.1% AcOH in MeOH	HESI in negative ion and positive ion modes	Thermo Scientific UHPLC	Exactive Hybrid Quadrupole‐Orbitrap	‐
Dincel et al. (2023)	AEA, 1‐AG, 2‐AG	‐	SPME with MeCN: i‐PrOH 1:1	‐	C18 (1.6 μm, 100 mm × 2.1 mm)	0.4 mL/min	0.3% FA in H_2_O	MeCN: i‐PrOH 90:10	ESI in positive ion mode	‐	Shimadzu 8060	131–181 ng/mL
Fanti (2023)	AEA, 2‐AG, OEA, PEA, NAGly, NASer, NAGABA, NAAla, NADA	Micro SPE with OMIX C18 pipette tips	Homogenised with 1:5 brain tissue: 0.2 M FA in MeOH	2 µL 10 mM FA in MeOH	C18 (1.7 μm, 100 mm × 2.1 mm)	0.3 mL/min	0.01% FA in H_2_O	0.01% FA in MeCN: MeOH 1:1	ESI in positive ion and negative ion modes	Sciex UHLPC	Sciex QTRAP 6500+	1–329 pg/g
Oliveira (2021)	AEA, 2‐AG	Protein precipitation with MeCN and 5 M NH₄HCO₂	Homogenised with 1:4.5 brain tissue: 0.1 M FA	10 µL 0.5% FA in H_2_O	C18	0.2 mL/min	H_2_O:MeCN 80:20	0.5% FA in H_2_O:MeCN 30:70	ESI in positive ion mode	Waters UHPLC	Waters Xevo TQ	6–10 ng/mL
Oliveira & Queiroz (2020)	AEA, 2‐AG	Protein precipitation with MeCN and 5 M NH₄HCO₂	Homogenised with 1:4.5 brain tissue: 0.1 M FA	10 µL 0.5% FA in H_2_O	C18 (1.7 μm, 100 mm × 2.1 mm)	0.4 mL/min	0.5% FA in H_2_O	0.5% FA in H_2_O:MeCN 30:70	ESI in positive ion mode	Waters Acquity UPLC	Waters Xevo TQ	2–300 ng/mL
Rand (2018)	AEA, EET‐EA, DHET‐EA	SPE with HLB Oasis cartridges	Homogenised with 400 µL 0.1% AcOH in MeOH	5 µL MeOH	C18 (1.7 μm, 150 mm × 2.1 mm)	‐	0.1 mM NH_4_CH_3_CO_2_and 0.1% FA in H_2_O	0.1 mM NH_4_CH_3_CO_2_ and 0.1% FA in MeCN	ESI in positive ion mode	Waters Acquity UPLC	Waters Xevo TQ	‐
Roszkowska (2023)	AEA, 2‐AG, NADA, 2‐AGe	‐	SPME with MeOH: i‐PrOH 1:1	10 µL i‐PrOH: MeOH 1:1	C18 (2.6 μm, 100 mm × 2.1 mm)	0.3 mL/min	0.1% FA in H_2_O	0.1% FA in MeCN	ESI in positive ion mode	‐	Shimadzu 8060	‐

Abbreviations: 1‐AG, 1‐arachidonoyl glycerol; 2‐AG, 2‐arachidonoyl glycerol; 2‐AGe, 2‐arachidonyl glyceryl ether; AEA, arachidonoyl ethanolamide; AcOH, acetic acid; DHET‐EA, dihydroxyeicosatrienoic acid ethanolamide; EET‐EA, epoxyeicosatrienoic acid ethanolamide; EMR, enhanced matrix removal; ESI, electrospray ionisation; FA, formic acid; HESI, heated electrospray ionisation; HLB, hydrophilic‐lipophilic‐balanced; i‐PrOH, isopropanol; LEA, linoleoylethanolamide; MeCN, acetonitrile; MeOH, methanol; NAAla, N‐arachidonoyl‐alanine; NADA, N‐arachidonoyl dopamine; NAGABA, N‐arachidonoyl‐γ‐aminobutyric acid; NAGly, N‐arachidonoyl‐glycine; NASer, N‐arachidonoyl‐serine; OEA, oleoylethanolamide; PEA, palmitoylethanolamide; VEA, vaccenoylethanolamide.

^#^ List of endocannabinoids analysed in this method is too long to list here.

^^^ Uses in vivo SPME.

Oliveira and Queiroz (2020) report homogenisation of brain samples with 0.2 M formic acid in methanol, following by protein precipitation of the homogenised supernatant. In this study, protein precipitation is conducted using 5 M ammonium formate in acetonitrile, and samples were subsequently analysed for AEA and 2‐AG using methods similar to the established methods that are described in detail elsewhere in this review. This research group extended this work to describe a novel method that uses in‐tube solid phase microextraction (SPME) coupled with restricted access materials in place of a liquid chromatography unit (Oliveira, 2021). It was found that this method achieved sufficient separation using in‐tube SPME to allow selectivity of AEA and 2‐AG, as well as sufficient method sensitivity to detect these endocannabinoids in rat brain samples (Oliveira, 2021). Further, both of these methods show excellent accuracy and precision, on par when compared to Ahmed (2022), albeit with higher LLOQs. Comparison of these methods also shows improved chromatographic resolution and separation of 1‐AG from 2‐AG in Ahmed (2022).

SPME has been increasingly favored as the primary sample preparation step by a number of recent articles. Beginning with Aslam (2019), SPME methods have been developed that avoid the limitation of conventional methods for endocannabinoids—being highly lipophilic—to bind to the tubing and probes that are used to extract the tissue samples (Arthur & Pawliszyn, 1990). Notably, Aslam (2019) apply their SPME technique in vivo, which presents promising new opportunities for measuring brain endocannabinoid activity in real time. However, compared to other methods this uses TripleTOF, which typically has a longer runtime and is more expensive. Sensitivity of this method was better than other methods reported, though has only acceptable (rather than excellent) intra‐session and inter‐session precision. Dincel, Zeinali, et al. (2023) also used SPME and found that use of 50:50 (v/v) methanol:isopropanol desorption solvent was optimal. This group applied phosphate‐buffered saline solution and a polydimethylsiloxane membrane coating to glass and plastic vials used during sample preparation, to avoid issues caused by the hydrophobic nature of endocannabinoids (e.g., poor water solubility, tendency to adsorb to plastic and glass consumables) (Dincel, Rosales‐Solano, et al., 2023; Dincel, Zeinali, et al., 2023). Compared to the other methods, this method lacks sensitivity and may not be sensitive enough for some applications. Imprecision values for 2‐AG and AEA were occasionally above 20%, indicating overall poorer performance compared to other methods reviewed in this section. Finally, Roszkowska (2023) replicated this method and expanded the targeted endocannabinoids to include N‐arachidonoyl‐dopamine as well as 2‐arachidonoyl glycerol ether (in addition to AEA and 2‐AG), which were both detectable with adequate sensitivity and reliability from brain samples using SPME for the first time. Validation parameters were not described in this study.

#### 3.2.1 Summary: The best methods for brain samples

Samples from brain tissue are preferably cleaned up using a form of SPE or protein precipitation and homogenized with an acetic acid mixture for extraction. Formic acid in water is favored for mobile phase A. Options for mobile phase B have remained exploratory, though usually include solutes of acetonitrile or methanol. As previously noted, ionisation in ESI positive ion mode in conjunction with a C18 analytical column (sub‐2 μm particle size) is recommended. This can successfully be achieved on a triple quadrupole mass spectrometer.

The future directions for this field appear to be around optimisation of sample preparation methods, such that new technologies for measuring endocannabinoid levels in brain tissue in vivo and effectively using standard methods are improved. Future work needs to balance effective methods for sample collection and processing that support experimental design with the necessity of maintaining high‐quality data. In some cases, these endeavors have not succeeded. Further, despite these advances in techniques, it can be seen from this review that there are distinct advantages for maintaining best practices in mass spectrometry methods, given that some methods still show high LODs and high variability of measurement. Use of high‐resolution instruments is not necessary.

### 3.3 Hair samples

The first analysis of endocannabinoids in human hair was performed by Krumbholz (2013). The method pioneered by these authors is frequently used in recent literature, and in Table 3 is used by Behnke (2021) to measure AEA, 2‐AG, OEA, PEA, and SEA in women with major depression. This method uses ball‐mill powdering and methanol extraction, followed by SPE, to prepare the samples. The now frequently used ammonium acetate mobile phase A was reported in this method, which has now been adopted across the hair and saliva endocannabinoid analysis literature.

**Table 3:** Analytical methods and parameters of recent methods for quantifying endocannabinoids in hair using LC‐MS.

Study	Analyte(s)	Wash	Extraction	Injection	Column	Flow Rate	Mobile Phase A	Mobile Phase B	Ionisation	LC System	MS System	LLOQ
Behnke (2021)	AEA, 2‐AG, OEA, PEA, SEA	Ball mill powdering	6 h sonication in 1.5 mL MeOH + SPE	‐	Agilent ZORBAX‐Eclipse XDB C8	‐	H_2_O:MeCN (95:5), 2 mM NH_4_CH_3_CO_2_, 0.1% AcOH	H_2_O:MeCN (5:95), 2 mM NH_4_CH_3_CO_2_, 0.1% AcOH	‐	Agilent HPLC 1290 infinity	Sciex TripleTOF 6600	1–100 pg/mg
Chu (2020)	AEA, 1‐AG	3 mL MeOH	24 h 1 mL MeOH + SPE	‐	C18 (5 μm, 150 mm × 4.6 mm)	0.5 mL/min	2 mM NH_4_CH_3_CO_2_ in MeOH: H_2_O (90:10)	‐	APCI in positive ion mode	Agilent HPLC 1200	Sciex QTRAP 3200	0.4–1.3 pg/mg
Gao (2020)	AEA, 2‐AG, OEA, PEA, SEA	2.5 mL C_3_H_8_O	18 h 1.8 mL MeOH + online SPE	100 µL MeOH: H_2_O 1:1	Shim‐pack XR‐ODS (2.2 μm, 75 × 3.0 mm)	‐	MeOH	2 mM NH_4_CH_3_CO_2_ in H_2_O	ESI in positive ion mode	Shimadzu UHPLC	Sciex QTRAP 6500	0.3–6 pg/mg
Ney et al. (2021)	AEA, 2‐AG, OEA	2 mL C_3_H_8_O: C₆H₁₄ (4:1, v/v)	18 h 1.6 mL MeOH	2 µL CHCI_3_	C18 (1.7 μm, 100 mm × 2.1 mm)	0.35 mL/min	2 mM NH_4_CH_3_CO_2_ in H_2_O	MeCN	ESI in positive ion mode	Waters Acquity UPLC	Waters Xevo TQ	0.4–5 pg/mg
Voegel (2021)	AEA, 2‐AG, OEA, PEA	15 mL H_2_O, 10 mL acetone	4 h sonication in 1 mL MeOH + SLE	10 µL 30% MeOH	C18 (2.6 μm, 50 mm × 2.1 mm)	0.4 mL/min	0.2 mM NH_4_F in H_2_O/MeOH 97:3	0.2 mM NH_4_F in H_2_O/MeOH 3:97	ESI in positive ion mode	Prominence UFLC	Sciex QTRAP 6500	0.5–2 pg/mg

Abbreviations: 2‐AG, 2‐arachidonoyl glycerol; AcOH, acetic acid; AEA, arachidonoyl ethanolamide; APCI, atmospheric pressure chemical ionization; C_3_H_8_O, isopropanol, C₆H₁₄, hexane; ESI, electrospray ionisation; H_2_O, water; MeCN, acetonitrile; MeOH, methanol; NH_4_CH_3_CO_2_, ammonium acetate; NH_4_F, ammonium fluoride; OEA, oleoylethanolamide; PEA, palmitoylethanolamide; SLE, supported liquid extraction.

The method that was developed after Krumbholz (2013) was that of Mwanza (2016). Comparison of the two methods shows that while the latter method avoided ball milling of hair samples, it retained a wash procedure (3 mL methanol) as well as methanol extraction of endocannabinoids, followed by SPE clean‐up of the samples. In contrast to the seminal study, the method developed by Mwanza (2016)—used by Chu (2020) as shown in Table 3—used atmospheric pressure chemical ionization. The authors report that this method was able to quantify 2‐AG, 1‐AG, and AEA in human and rodent hair samples (Chu, 2020; Mwanza, 2016) at a lower concentration compared to the seminal method. This suggests that extensive sample preparation (ball‐milling of hair samples) is not necessary.

Our group developed a similar method that was used to quantify AEA, 2‐AG, and OEA in human hair (Ney, 2021a). This method used ESI in a triple quadrupole mass spectrometer, though retained the alcoholic wash step and methanol extraction reported by the previous studies. In contrast to the previous work, we found that hair sample methanol extracts, once evaporated to dryness, were frequently not water soluble and therefore not suitable for SPE. Our method therefore did not use any clean‐up step beyond initial methanol extraction, though required chloroform resuspension before spectrometer injection for adequate chromatography (Ney, 2021a). Compared to Chu (2020) we found a similar LOD for all analytes, again suggesting that extensive sample preparation (SPE) is not necessary when dealing with hair samples. Interestingly, when changing the device used for solvent evaporation from a nitrogen stream thermal block to a vacuum evaporator, we have been able to resuspend samples in a more acceptable solution, such as acetonitrile or acetonitrile and water (1/1, v/v). This method has been applied to research seeking to understand the relationship between endocannabinoids and fear learning in humans (Ney, Crombie, et al., 2023; Ney, Nichols, et al., 2023), which is relevant to posttraumatic stress and anxiety disorders.

Two other recent methods have also found that offline SPE is not essential to quantification of endocannabinoids in hair (Gao, 2020; Voegel, 2021). Notably, both of these methods avoid ball‐milling by using simple alcoholic washing steps, followed by methanol extraction of lipids from the samples. Both methods also use a SCIEX 6500 QTRAP with ESI in positive ion mode and do not require sub‐2 µm particle size C18 columns to achieve similar sensitivity compared to our method (Ney, 2021a). When comparing these methods, two key differences in the methods are evident. First, while Gao (2020) uses ammonium acetate‐based mobile phases, Voegel (2021) substitutes this for ammonium fluoride. We have recently made a similar substitution in our laboratory and found that ammonium fluoride results in better sensitivity for steroid hormones, though slightly decreased peak resolution for endocannabinoids. This may be due to ammonium fluoride preventing the formation of M+ sodium species by binding free sodium and forming sodium fluoride (McFadden & Ames, 2023). Second, while Voegel (2021) uses offline‐supported liquid extraction, Gao (2020) uses online SPE. Compared to our study (Ney, 2021a), both methods display improve peak shape during analysis, though both have a longer runtime (19+ min compared to 12 min for our method). Both methods have reported similar limits of detection and quantification for endocannabinoids in human hair and have since been applied in numerous studies examining the relationship between endocannabinoids and various psychological characteristics (Croissant, 2020; Gao, 2022; Hitzler, 2023; Koenig, 2018; Mourtakos, 2021; Mytareli, 2023; Planert, 2023; Tam, 2021; Valdivieso Barba et al., 2023; Voegel, 2022; Walther, 2023; Wingenfeld, 2018). Interestingly, hair endocannabinoids were found to not correlate with plasma or urine endocannabinoid levels (Valdivieso Barba et al., 2023), though showed strong test–retest value over a period of two and a half years (Gao, 2021). Plasma, urine, and hair samples may reflect independent ECB levels that individually relate to the function of their system, instead of the circulating levels (Valdivieso Barba et al., 2023). Relatedly, ECBs play an important role in intercellular communication in an endocrine fashion (Jafarpour et al., 2017; Pagotto, 2006; Piomelli, 2003), suggesting that the synthesis, release, and regulation of ECBs may not necessarily correlate to other organs, tissues, or excretion productions (like plasma, urine, or hair), but only to other biological signalling systems, such as the stress response via glucocorticoids.

#### 3.3.1 Summary: The best methods for hair samples

Highly efficient hair analysis methodologies are now available for the quantification of endocannabinoids using LC‐MS/MS. These methods have begun to be applied widely across psychiatric disorders and have some distinct advantages over other peripheral measures due to ease of collection and long‐term retrospective analysis, owing to the slow growth of hair. Comparison between the available methods suggests that overnight methanol extraction of analytes has been reported to be sufficient and SPE does not appear to be necessary following extraction. Multiple studies report method efficiency using ammonium acetate in water as mobile phase A, though ammonium fluoride might also be appropriate, where laboratory conditions permit. As with other endocannabinoid methods, a C18 column and electrospray ionisation in positive ion mode are appropriate. Sub‐picogram per milligram concentrations of hair endocannabinoids should be achieved and hair endocannabinoids are reported in this range even using older model mass spectrometers with an appropriate method. Current challenges for the field include improving efficiency of analysis runtime while maintaining data quality. The biological role of endocannabinoids present in hair needs clarification before it can be established as a useful biomarker. Existing studies have shown inconsistencies in the ability of hair endocannabinoids to reliably predict psychological characteristics (Croissant, 2020; Gao, 2022; Hitzler, 2023; Koenig, 2018; Mourtakos, 2021; Mytareli, 2023; Planert, 2023; Tam, 2021; Valdivieso Barba et al., 2023; Voegel, 2022; Walther, 2023; Wingenfeld, 2018).

### 3.4 Saliva samples

Endocannabinoids have been quantifiable using LC‐MS methods in human saliva since 2012 (Table 4) (Matias, 2012). Our group has validated that a simple LC‐MS/MS method is sufficient to detect AEA, 2‐AG, and OEA at extremely low levels in saliva (Ney, 2020), and we have used this technique to show that endocannabinoids in saliva are stress‐reactive (Ney, Crombie, et al., 2021; Ney, Crombie, et al., 2023). Our method uses 50:50 methanol:acetone protein precipitation and liquid extraction, which is added to 400 µL of saliva (a higher volume is necessary to increase method sensitivity). This method requires an aqueous solution of ammonium acetate as mobile phase A to achieve separation of 1‐AG from 2‐AG, as well as to achieve adequate sensitivity. In particular, modern instrumentation is required for detection of AEA in saliva, which tends to be present at low pg/mL concentrations. Conversely, OEA is highly abundant, and 2‐AG is abundant enough to quantify in most samples (Ney, 2021b). Though, specific diseases such as Huntington's Disease have been associated with downregulated ECBs (Micale et al., 2007), whereby levels of circulating ECBs may be lower than the LLOQs. Samples are analysed using a triple quadrupole mass spectrometer operating in positive ionisation mode (Ney, 2020).

**Table 4:** Analytical methods and parameters of recent methods for quantifying endocannabinoids in saliva using LC‐MS.

Study	Analyte(s)	Extraction	Injection	Column	Flow Rate	Mobile Phase A	Mobile Phase B	Ionisation	LC System	MS System	LLOQ
De Luca (2020)	OEA, PEA, LEA, NAPE	B&D method	10 µL CH_3_CN:C_3_H_8_O:H_2_O (60:35:5)	C18 (2.6 μm, 150 mm × 2.1 mm)	0.2 mL/min	5 mM NH₄HCO₂, 0.1% FA in 40:60 CH_3_CN/H_2_O	5 mM NH₄HCO₂, 0.1% FA in 90:10 C_3_H_8_O/CH_3_CN	HESI in positive ion and negative ion modes	Accela UHPLC	Exactive mass spectrometer	‐
Haviv (2022)	AEA, 2‐AG, OEA, PEA, AA	Acetone,# B&D method	‐	C18 (2.6 μm, 150 mm × 2.1 mm)	‐	‐	‐	‐	Shimadzu UHPLC	Sciex 5500 QTRAP	‐
Mennella (2018)	AEA, 2‐AG, OEA, PEA, LEA	Acetone,# B&D method	20 µL 1:1 MeCN: H_2_O	RP 80	0.2 mL/min	‐	‐	ESI or APCI in positive ion mode	Perkin‐Elmer series 200	API 3000	‐
Ney et al. (2020)	AEA, 2‐AG, OEA	Acetone#: MeOH 1:1, v/v	15 µL MeOH: H_2_O	C18 (1.7 μm, 100 mm × 2.1 mm)	0.35 mL/min	2 mM NH_4_CH_3_CO_2_in H_2_O	MeCN	ESI in positive ion mode	Waters Acquity UPLC	Waters Xevo TQ	4–280 pg/mL
Tarragon (2020)^	AEA, 2‐AG, PEA, AA	‐	‐	‐	‐	‐	‐	‐	‐	‐	‐
Tinto (2021)	13‐HODE‐EA	B&D method	40 µL 1:1 Solvent A: Solvent B	C8 (2.6 μm, 150 mm × 2.1 mm)	0.4 mL/min	1 mM NH₄OH, 0.5% AcOH in H_2_O	1 mM NH₄OH, 0.5% AcOH in 95% MeCN	ESI in positive ion mode		Shimadzu 8050	25 fmol
Wu (2020)	AEA	Ethyl acetate#	20 µL Solvent A	C18 (5 μm, 150 mm × 4.6 mm)	0.5 mL/min	4 mM NH_4_CH_3_CO_2_in 95% MeOH	‐	APCI in positive ion mode	Agilent 1200 HPLC	Sciex 3200 QTRAP	12 pg/mL

Abbreviations: 2‐AG, 2‐arachidonoyl glycerol; 13‐HODE‐EA, 13‐hydroxy‐9Z,11E‐octadecadienoyl‐N‐ethanolamine; AA, arachidonic acid; AcOH, acetic acid; AEA, arachidonoyl ethanolamide; APCI, atmospheric pressure chemical ionization; B&D, Bligh and Dyer (Bligh & Dyer, 1959); ESI, electrospray ionisation; FA, formic acid; HESI, heated electrospray ionisation; LEA, linoleoylethanolamide; MeCN, acetonitrile; MeOH, methanol; NAPE, N‐arachidonoylphosphatidylethanolamine; OEA, oleoylethanolamide; PEA, palmitoylethanolamide.

^#^ These methods performed protein precipitation liquid extraction.

^^^ This study did not report analysis methods or parameters.

Other recent studies have demonstrated that other methods can be used to quantify endocannabinoids in saliva. Tarragon (2020) reported using a method that was a mix between the approaches for analysing endocannabinoids in blood samples that were adopted by Fanelli (2012), who used toluene extraction, and Bindila and Lutz (2016), who used ethyl acetate:hexane extraction. Notably, Fanelli (2012) found that toluene (compared to ethyl acetate:hexane) resulted in superior extraction and reduced isomerisation of 2‐AG. Tarragon (2020) reported that body composition and omega 6:omega 3 fatty acid ratio was predicted by salivary endocannabinoid concentrations in women consuming high processed foods. Ethyl acetate precipitation was also used by Wu (2020), in conjunction with an aqueous ammonium acetate mobile phase followed by atmosphere pressure chemical ionization to quantitate AEA.

Saliva analysis has similarly been recently applied to understanding endocannabinoid activity during food intake and mastication. Mennella (2018) used a protocol adapted from Kong (2016), where 1 mL of saliva was purified using HLB SPE cartridges, extracted with chloroform:methanol, and analysed with a HPLC system coupled with a triple quadrupole mass spectrometer. The same research group later used chloroform:methanol extraction followed by high‐resolution mass spectrometry to test saliva samples for OEA, PEA, and LEA, as well as N‐arachidonoylphosphatidylethanolamine (NAPE) (De Luca, 2020). Although successful for these targets, this method was not reported to analyse AEA or 2‐AG.

Similar to Ney (2020), acetone protein precipitation (with 50 mM Tris buffer), followed by chloroform:methanol extraction, was employed by Haviv (2022) to measure AEA, 2‐AG, PEA, and OEA in saliva. Similar processing conditions included use of a sensitive triple quadrupole mass spectrometer and C18 analytical column. This group also found that salivary OEA, 2‐AG, and AEA were significantly lower in patients with orofacial pain compared to healthy controls (Haviv, 2022).

Interestingly, a recent study found evidence that a novel endocannabinoid, 13‐hydroxy‐9Z,11E‐octadecadienoyl‐N‐ethanolamine (13‐HODE‐EA), was a biosynthetic product of LEA and was present in human saliva and skin (Tinto, 2021). LC‐MS/MS analysis of 13‐HODE‐EA was achieved in saliva with chloroform:methanol extraction and analysis using a triple quadrupole mass spectrometer paired with a HPLC system using a C8 column with acidified aqueous and organic mobile phases containing ammonium hydroxide (Tinto, 2021).

#### 3.4.1 Summary: The best methods for saliva samples

The advent of modern LC‐MS/MS instrumentation has enabled the analysis of endocannabinoids in saliva by laboratories around the world. While methods differ slightly, usually organic liquid extraction (either chloroform partitioning or acetone protein precipitation) appears to be sufficient for sample preparation, and use of binary HPLC, C18 column setups coupled with a sensitive triple quadrupole mass spectrometer for analysis are sufficient for endocannabinoid quantification at low pg/mL levels. While the literature is still in its infancy, low additions of ammonium acetate to water have been reported to be efficacious as mobile phase A. Due to underreporting in this literature, it is not possible to directly compare different LC‐MS/MS methods for endocannabinoids in saliva.

As an emerging field, there are multiple improvements that need to be made. First, the origin and function of salivary endocannabinoids, as well as their responsivity to various environmental stimuli, need to be clarified before they can be useful as biomarkers. LC‐MS/MS methods can be improved by increasing sensitivity, particularly to low abundance analytes such as AEA, which, until very recently, was undiscoverable in saliva. Future studies that examine more optimised sample clean‐up may be able to improve sensitivity towards AEA. This might be achievable through learning lessons from other fields—for example, the routine use of sub‐2 µm particle columns and the addition of higher‐risk solvents during sample preparation and analysis such as ammonium fluoride and toluene, which may improve sensitivity. More studies are also needed to provide independent validation of LC‐MS/MS methods for salivary endocannabinoids, so that they can be used in a range of settings, rather than a select and limited number of laboratories around the world. This might be achievable with the advent of more sensitive methods that make routine laboratories more likely to be able to detect AEA in saliva.

### 3.5 Cerebral spinal fluid

The first published method for the quantification of endocannabinoids in cerebrospinal fluid (CSF) was developed by Giuffrida (2004) in 2004, though the assay was only an adaptation of a previously published method in plasma (Giuffrida et al., 2000a). CSF samples were collected using a nontraumatic lumbar puncture procedure, and aliquots of 1 mL were spiked with 25 pmol of internal standards of AEA, PEA, OEA, and proteins were precipitated with acetone. The supernatants were evaporated and partitioned with 2:1 chloroform:methanol. AEA, PEA, and OEA were quantified using a C18 column and a HP 1100 Series HPLC/MS system. The LLOQs were reported as 0.3 pmol for AEA and 0.1 pmol for PEA and OEA.

In recent years, only a handful of assays quantifying endocannabinoids in CSF have been published (Table 5). The CSF samples remain to be commonly collected with a nontraumatic lumbar puncture procedure, typically around midday, and immediately stored at −80°C. Though, with assays designed specifically for analytes in CSF, a wider range of targets with low LLOQs have been reported. For example, Aydin (2023) extracted 1‐AG, 2‐AG, 2‐AG ether, AEA, LEA, PEA, and OEA from CSF using simple protein precipitation and homogenisation with 50 μL acetonitrile:water mixture, followed by a 2 min sonication. The endocannabinoids were analysed with an Agilent 1290 Infinity II liquid chromatography system and a QTRAP 4500 mass spectrometer equipped with a C18 column. The analytes were eluted on a gradient of water and acetonitrile with a constant concentration of 0.1% formic acid. The LLOQs were reported in a range from 0.378 to 9.114 pM.

**Table 5:** Analytical methods and parameters of recent methods for quantifying endocannabinoids in cerebral spinal fluid using LC‐MS.

Study	Analyte(s)	Extraction	Injection	Column	Flow Rate	Mobile Phase A	Mobile Phase B	Ionisation	LC System	MS System	LLOQ
Ayden et al. (2023)	1‐AG, 2‐AG, 2‐AGE, AEA, LEA, PEA, OEA	Protein precipitation, homogenised with 50 µL MeCN:H_2_O, and 2 min sonication	20 µL	C18 (2.7 µm, 50 mm × 2.1 mm)	0.2 mL/min	H₂O with 0.1% FA	MeCN with 0.1% FA	ESI in positive ion mode	Agilent 1290 Infinity II LC	QTRAP 4500 mass spectrometer	0.378–9.114 pM
He (2022)	LEA, EPEA, POEA, PDEA, DHEA, AEA, LEA, 1‐AG/2‐AG, 1‐LG/2‐LG, PEA, ETAEA, OEA, DEA, 1‐OG/2‐OG, SEA	Homogenisation and centrifugation with 1 mL MBTE:50 μL 0.1 M ammonium acetate:10 μL ISTD working solution; evaporated and reconstituted with 20 μL H_2_O:MeCN, then centrifuged	3 μL	C18 (2.6 μm, 150 mm × 0.3 mm)	4 μL/min	2 mM NH_4_HCO_2_ with 10 mM FA in H₂O	MeCN	ESI in positive ion mode	Waters nanoAcquity LC	Shimadzu LCMS‐8060 triple quadrupole	2.0–152.4 pM (and 4311.3 pM for PEA)
Kantae (2017)	PEA, LEA, OEA, SEA, AEA, DGLEA, DHEA, DEA, 1‐AG/2‐AG	Homogenisation and centrifugation with 100 μL 100 nM citrate buffer:25 nM EDTA:10 μL deuterated ISTD solution; evaporated and reconstituted with 10 μL MeOH:H_2_O	8 μL	C18 (3 μm, 150 mm × 0.75 mm)	2 μL/min	10 mM FA/H₂O	MeCN	ESI in positive ion mode	Agilent 1100/1200 series nano‐LC	Agilent 6460 triple quadrupole	0.86–107.4 pM (and 445 pM for SEA)
Oliviera et al. (2019)	AEA	Disposable pipette extraction (SPE)	10 μL	C18 (1.7 μm, 100 mm × 2.1 mm)	0.4 mL/min	0.5% FA in H₂O	MeCN	ESI in positive ion mode	Waters ACQUITY UPLC H‐Class	Xevo TQ‐D triple quadrupole	0.100 ng/mL

Abbreviations: AEA, anandamide; AG, arachidonoylglycerol; DEA, docosatetraenoyl ethanolamide; DGLEA, dihomo‐γ‐linolenoyl ethanolamide; DHEA, docosahexaenoyl ethanolamide; EDTA, ethylenediaminetetraacetic acid; EPEA, eicosapentaenoyl ethanolamide; ESI, electrospray ionisation; ETAEA, mead acid ethanolamide; FA, formic acid, H₂O, water; ISTD, internal standard; LEA, linoleoyl ethanolamide; LG, linoleoyl glycerol; MBTE, methyl tert‐butyl ether; MeCN, acetonitrile; NH_4_HCO_2_, ammonium formate; OEA, oleoylethanolamine; OG, oleoyl glycerol; PDEA, pentadecanoyl ethanolamide; PEA, palmitoyl ethanolamide; POEA, palmitoleoyl ethanolamide; SEA, stearoyl ethanolamide.

He (2022) developed a micro‐LC‐MS assay for the quantification of AEA, LEA, 1‐AG, 2‐AG, PEA, OEA, and many related endocannabinoids. To extract the analytes from CSF, 10 μL of the internal standard working solution was homogenized and centrifuged with 1 mL MBTE and 50 μL 0.1 M ammonium acetate buffer solution. After centrifugation, the organic supernatant was evaporated with water:acetonitrile. The endocannabinoids were analysed with a Waters nanoAcquity liquid chromatography system and a Shimadzu 8060 triple quadrupole equipped with a C18 column. With a larger analyte list, most LLOQs were reported at 2.0 to 152.4 pM, along with an LLOQ of 4311.3 pM for PEA. Compared to Aydin (2023), this method shows poorer sensitivity, suggesting that either microflow is inefficient for endocannabinoid quantification or that the injection volume relative to the final sample concentration was too low. Similarly, Kantae (2017) homogenized and centrifuged 10 μL of a deuterated internal standard solution consisting of PEA, OEA, AEA, 1‐AG, 2‐AG, and related endocannabinoids with 100 μL 100 nM citrate buffer, 25 nM EDTA, and 550 μL toluene. The supernatant was evaporated and reconstituted with water and methanol. The endocannabinoids were analysed with an Agilent 1100/1200 series nano‐liquid chromatographer and 6460 triple quadrupole equipped with a C18 column. The analytes were eluted in a gradient with 2 mM ammonium formate with 10 mM formic acid in water, and acetonitrile. Most LLOQs for the analytes were reported in a range from 0.86 to 107 pM, along with 445 pM for N‐stearoylethanolamine. Compared with He (2022), this method demonstrates that microflow was not the cause of the high LODs, though of all the methods reviewed in this section He (2022) achieved the best peak resolution during analysis, despite the loss of sensitivity for some analytes.

Oliveira et al. (2019) published the most recent assay quantifying AEA in CSF using SPE as the extraction method. Disposable Pipette Extraction (DPX) is a form of SPE procedure involving a 1 mL DPX tip containing 60 mg of C18 phase freely accommodated between two porous polymer filters. The filter is washed with a 60:40 water:acetonitrile solution in two draw/eject cycles. The CSF sample was drawn into the pipette tip with an attached syringe device follow by turbulent air bubble aspiration to create a sorbent suspension. AEA is eluted with acetonitrile in two draw/eject cycles, which is then evaporated and reconstituted with the mobile phase. AEA was then analysed in a Waters ACQUITY UPLC H‐Class liquid chromatographer and a Xevo TQ‐D tandem quadrupole with a C18 column, with an LLOQ of 0.100 ng/mL. Compared to the previously mentioned methods, this does not achieve impressive sensitivity, given that all other methods for CSF reviewed in this article achieve sensitivity in the low pg/mL range for AEA.

#### 3.5.1 Summary: The best methods for cerebral spinal fluid

Only a few new LC‐MS/MS methods for quantifying endocannabioniods have been developed for CSF since 2017—suggesting robustness of existing methodology—though some emerging preferences and recommendations can be noted. Extraction of the analytes from the biomatrix is usually performed by protein preciptation using water and acetonitrile or methanol. The only method using SPE reported a much higher LOD compared to methods not using SPE. Formic acid in water as mobile phase A is unequivocably preferred, along with acetonitrile as mobile phase B. Large target analyte lists are usually invesitgated in cerebral spinal fluid, with analysis on a triple quadropule mass spectrometer for increased specificity. On every occasion, ionisation has been reported as ESI in positive ion mode, in conjunction with a C18 analytical column. Sub‐2 µm particle size columns are not necessary for CSF endocannabinoid measurement.

This literature is still emerging, but it is clear that endocannabinoids in CSF can be routinely and adequately measured using existing techniques and technologies. A challenge highlighted by this field, however, is that high N‐acyl ethanolamide levels can occur during sample preparation and method execution. Kantae (2017) report that, in their study, inflated LLOQ for SEA was due to a contaminant peak present in ethanol added during sample collection, reinforcing the pervasive nature of N‐acyl ethanolamides in standard laboratory materials. This issue presents a general limitation of N‐acyl ethanolamide measurement and is something that needs to be addressed in future studies examining the exact laboratory conditions necessary for specific analysis of these molecules.

### 3.6 Other biological sample matrices

As can be seen in Table 6, MS methods are consistent across different biological matrices, and these same methods apply throughout biomatrices detailed in this review. Most studies use positive ion electrospray ionisation with multiple reaction monitoring as the triple quadrupole mode of operation, though for some targets (such as arachidonic acid), negative ionisation mode is also used (Casati, 2020; Kayacelebi, 2017). Typically, aqueous mobile phase (A) is used, usually with the addition of either acetic acid, ammonium acetate, formic acid, or ammonium fluoride. Solvents such as acetonitrile or methanol are usually used as mobile phase B. C18 columns have been exclusively used in this literature.

**Table 6:** Analytical methods and parameters of recent methods for quantifying endocannabinoids in other biological sample matrices using LC‐MS.

Study	Analyte(s)	Matrix	Extraction	Injection	Column	Flow Rate	Mobile Phase A	Mobile Phase B	Ionisation	LC System	MS System	LOQ
Bobrich (2020)	AEA, 2‐AG, NADA, 2‐Age, VA	Human lung cancer cells	ethyl acetate	80 µL 0.2% FA in MeCN: H_2_O 60:40	C18 (5 µm, 250 mm × 2 mm)	0.3 mL/min	0.2% FA in H_2_O	0.2% FA in MeCN	ESI in positive ion mode	Waters HPLC 2695	Micromass Quattro Micro API	0.03–2 ng/mL
Casati (2020)	AEA, OEA, LNEA, PEA, LEA, SEA, 2‐AG, Ogly, Aser, DHEA, EPEA, ADA, ODA, Agly, A5HT, 2Age, AGABA, AA, Pal5HT, O5HT	Saos‐2 and MG‐63 cells	4 mL of CH_2_Cl_2_: i‐PrOH 80:20	3 µL MeOH	C18 (2.6 µm, 150 mm × 2.1 mm)	0.6 mL/min	0.1% FA in H_2_O	MeOH: MeCN 5:1	ESI in positive ion and negative ion modes	Agilent 1290 Infinity UHPLC	Sciex QTRAP 16 5500	15–109 pg/mL
Datta (2021)	AEA, 2‐AG	Human milk	‐	‐	C18 (3 µm, 50 mm × 2 mm)	0.5 mL/min	‐	‐	ESI in positive ion mode	Sciex UHPLC	Sciex QTRAP 5500	‐
Fernández de Luco (2019)	2‐AG	*Caenorhabditis elegans*	0.5 M KCl/0.08 M H_3_PO_4_: CHCI_3_ 2:1	‐	‐	‐	H_2_0	MeCN	ESI in positive ion mode	‐	‐	5–16 ng/mL
Ding (2017)	AEA, 2‐AG, OEA, PEA	Human follicular fluid	MLME with toluene and 4‐DMABC derivatization	5 µL MeCN: H_2_O 7:3	C18 (1.7 µm, 100 mm × 2.1 mm)	0.3 mL/min	0.1% FA in H_2_O	0.1% FA in MeCN	ESI in positive ion mode	Shimadzu LC‐30AD UHPLC	Shimadzu 8040	10–181 fmol
Kayacelebi (2017)	AA	Dog liver and human monocytic cells	ethyl acetate	‐	C18 (1.7 µm, 100 mm × 2.1 mm)	0.4 mL/min	H_2_0	MeCN	ESI in negative ion mode	Waters Acquity UPLC	Waters Xevo TQ	<0.1 µM
Paquot et al. (2023)	AEA, 2‐AG, AA	Cervical tissue and J774 cells	Homogenised with CHCI_3_, MeOH, H_2_0. SPE with CHCI_3_ elution	2 µL MeOH	C18 (1.7 µm, 150 mm × 2.1 mm)	‐	0.1% AcOH in H_2_0: MeCN 95:5	0.1% AcOH in MeCN	ESI in positive ion and negative ion modes	Waters Acquity UPLC	Waters Xevo TQ	3–400 fmol
Restin (2022)	AEA, 2‐AG, OEA, PEA	Human nails	Ball‐milled, 1 mL MeOH, SLE with ethyl acetate	10 µL MeOH	C18 (2.6 µm, 50 mm × 2.1 mm)	0.4 mL/min	0.2 mM NH_4_F in H_2_0: MeOH 97:3	0.2 mM NH_4_F in H_2_0: MeOH 3:97	ESI in positive ion mode	Prominence UFLC	Sciex QTRAP 6500+	0.3–5 pg/mg
Richardson (2020)	AEA, 2‐AG, OEA, PEA	Human aqueous humour	Ethyl acetate:hexane 9:1	5 µL MeCN	C18 (1.7 µm, 150 mm × 2.1 mm)	0.45 mL/min	1 M NH_4_CH_3_CO_2_, 0.1% FA in H_2_O	1 M NH_4_CH_3_CO_2_, 0.1% FA in MeCN: H_2_O 9:1	ESI in positive ion mode	Sciex Exion UHPLC	Sciex QTRAP 6500+	‐
Sjodin et al., 2018	AEA, PEA, LEA, DHEA, OEA, 2‐AG	Human bronchoalveolar lavage fluid	SPE with Oasis HLB cartridges	‐	C18 (1.7 µm, 150 mm × 2.1 mm)	0.3 mL/min	0.1% AcOH in H_2_O	MeCN: i‐PrOH 9:1	ESI in positive ion mode	Waters Acquity UPLC	Waters Xevo TQ	‐
Svobodova (2023)	AEA, OEA, PEA	Human placental membranes	Homogenised in MeCN, extracted with MeCN/ethyl acetate, SPE with Oasis HLB	5 µL MeCN	C18 (1.7 µm, 50 mm × 2.1 mm)	0.7 mL/min	1 M NH_4_CH_3_CO_2_, 0.1% FA in H_2_O	0.1% FA in MeCN	ESI in positive ion mode	Sciex ExionLC UHPLC	Sciex QTRAP 6500+	‐
Tagliamonte (2021)	AEA, 2‐AG, OEA, PEA, LEA, SEA, NAPE	Human intestinal lumen	B&D method	10 µL MeCN: i‐PrOH: H_2_O 60:35:5	C18 (2.6 µm, 100 mm × 2.1 mm)	0.2 mL/min	5 mM NH₄HCO₂, 0.1% FA in H_2_O: MeCN 40:60	5 mM NH₄HCO₂, 0.1% FA in i‐PrOH: MeCN 90:10	HESI in positive ion mode	Accela U‐HPLC	Thermo Fisher Exactive Orbitrap	0.25–200 ng/mL
Valastro (2017)	AEA, 2‐AG, PEA, OEA	Dog synovial fluid	B&D method	‐	‐	‐	‐	‐	APCI	Shimadzu HPLC 10ADVP	Shimadzu 2020	‐
Zawatsky (2021)	AEA, 2‐AG	Mouse skin tissue	Homogenised with PCA, B&D method, acetone protein precipitation	‐	C18	0.25 mL/min	0.1% FA in H_2_O	0.1% FA in MeOH	ESI in positive ion mode	Agilent 1200 LC	Agilent 6410 triple quad	‐
Zufferey (2020)	AEA, 2‐AG, PEA, OEA	Human semen	MeCN and heptane:ethyl acetate	15 µL 0.1% FA in MeCN: H_2_0 65:35	RP‐18e 100‐3 column	0.4 mL/min	0.1% FA in H_2_O	0.1% FA in MeCN	HESI in positive ion mode	Thermo Scientific LC Accela 1200	Thermo Scientific TSQ Vantage	‐

Abbreviations: 2‐AG, 2‐arachidonoyl glycerol; 2‐Age, 2‐arachidonoylglyceryl ether; A5HT, arachidonoylserotonine; AA, arachidonic acid; AcOH, acetic acid; ADA, arachidonoyldopamine; AEA, arachidonoyl ethanolamide; AGABA, arachidonoyl‐3‐hydroxy‐γ‐aminobutyric acid; Agly, arachidonoylglycine; APCI, atmospheric pressure chemical ionization; Aser, arachidonoylserine; B&D, Bligh and Dyer (Bligh & Dyer, 1959); CHCI_3_, chloroform; DHEA, docosahexaenoylethanolamide; 4‐DMABC, 4‐(*N*,*N*‐dimethyamino)benzoyl chloride; EPEA, eicosapentaenoylethanolamide; ESI, electrospray ionisation; FA, formic acid; HLB, Hydrophilic‐Lipophilic‐Balanced; i‐PrOH = isopropanol, LEA, linoleoylethanolamide; LNEA, linolenoylethanolamide; MeCN, acetonitrile; MeOH, methanol; MLME, magnetic liquid microextraction; NADA, N‐arachidonoyl dopamine; NAPE, N‐acylphosphatidylethanolamine; O5HT, oleoylserotonine; ODA, oleoyldopamine; OEA, oleoylethanolamide; Ogly, oleoylglycine; Pal5HT, palmitoylserotonine; PalGly, palmitoylglicine; PCA, perchloric acid; PEA, palmitoylethanolamide; SEA, stearoylethanolamide; SLE, supported liquid extraction; VA, O‐arachidonoylethanolamine.

The key differences between these methods are therefore how the samples are prepared. Bobrich (2020) used ethyl acetate extraction for the analysis of endocannabinoids in human lung cancer cells, whereas Casati (2020) used a dichloromethane:isopropanol solution for endocannabinoid extraction from Saos‐2 and MG‐63 cells. Notably, both of these articles reported successful analysis of a wide range of endocannabinoids and N‐acylethanolamines. Similarly, Kayacelebi (2017) adopted ethyl acetate extraction for human monocytic cells, whereas Paquot et al. (2023) used a two‐step process involving chloroform:methanol separation (Bligh & Dyer, 1959) followed by SPE of the chloroform layer.

A wide range of human and animal biofluids have also been examined. Datta (2021) quantified AEA and 2‐AG in human milk, though did not report sample treatment protocols before analysis. Ding (2017) extracted endocannabinoids from human follicular fluid using magnetic iron oxide nanoparticles in toluene—previously reported to be the most efficient extraction solvent for endocannabinoids (Fanelli, 2012)—before performing 4‐(*N*,*N*‐dimethyamino)benzoyl chloride derivatisation. To our knowledge, this is the only study to use chemical derivatisation of endocannabinoids before analysis with LC‐MS/MS, and the authors reported substantial improvements in lower limits of detection using this method (Ding, 2017). However, it should be noted that this review suggests that endocannabinoids are easily quantifiable across a range of biomatrices without derivatization. Richardson (2020) used ethyl acetate and hexane (9/1, v/v) to extract endocannabinoids from human aqueous humour fluid, and similarly acetonitrile followed by ethyl acetate and heptane (1/1, v/v) extraction was used for semen samples by Zufferey (2020). Finally, while the Bligh and Dyer (1959) method was used for endocannabinoid extraction from human intestinal lumen (Tagliamonte, 2021) and dog synovial fluid (Valastro, 2017), endocannabinoids from human bronchoalveolar lavage fluid were extracted using Oasis Hydrophilic‐Lipophilic‐Balanced SPE cartridges (Sjödin, 2018).

Finally, endocannabinoids have been quantified using LC‐MS/MS in various human and animal tissues; again, the primary differences between the methods are related to sample preparation rather than the LC‐MS methods themselves. In one study, human cervical tissue was homogenised and extracted first using the Bligh and Dyer (1959) method followed by SPE (Paquot et al., 2023). In contrast, human placental membranes were extracted with sufficient efficiency using acetonitrile, ethyl acetate, and SPE (Svobodova, 2023), though the former study may have required more complete lipid separation due to measurement of prostaglandins in addition to endocannabinoids. Zawatsky (2021) homogenised mouse skin tissue samples with perchloric acid, before using Bligh and Dyer (1959) extraction followed by acetone protein precipitation in place of SPE. Finally, human nails were prepared using a ball‐mill and endocannabinoids were extracted using methanol and ethyl acetate (Restin, 2022).

#### 3.6.1 Summary: The best methods for other biological sample matrices

Endocannabinoids and related lipid compounds are quantifiable in a wide range of biomatrices using typical LC‐MS/MS methods. As previously reported, mobile phase A usually consists of formic acid in water, while mobile phase B uses formic acid in acetonitrile or methanol. While a C18 column is preferred and recommended, ionisation, extraction, and injection volume must be particular to the biomatrix, where further recommendations can be found in specific literature. In particular, researchers interested in analysis of endocannabinoids in atypical biomatrices should consult the provided resources to ensure that appropriate lipid extraction and sample clear‐up is performed before analysis.

### 3.7 Mass spectrometry imaging (MSI)

The following section describes emerging literature that, while ineligible for our systematic review, was frequently rejected during our search but is relevant for endocannabinoid analysis in biomatrices. This section is not exhaustive but serves as a precursor for emerging mass spectrometry applications within this field.

MSI is an emerging analytical technique that combines the capabilities of mass spectrometry and spatial mapping. MSI enables simultaneous analysis of the molecular composition of samples while preserving their spatial distribution. In MSI, a laser or flow of charged particles are used to desorb and ionize molecules from the sample surface, and the resulting ions are analysed based on their mass‐to‐charge ratios. MSI offers exciting possibilities for researchers in biological fields and has begun to be adopted with improving and increasingly affordable technologies, such as matrix‐assisted laser desorption ionisation (MADLI) and desorption electrospray ionisation (DESI) platforms.

Recent studies have shown that MSI is capable of assessing endocannabinoid concentrations across the brain. Using MALDI, at least three lipid species that likely consisted of arachidonic acid were reported to be expressed at reduced levels in mouse brains (cortex, cerebellum, hippocampus, and amygdala) from a transgenic depletion model of Alzheimer's Disease (González de San Román, 2021). Using DESI, 2‐AG was found to be at the highest concentrations in the hypothalamus and at the lowest concentrations in the hippocampus (Zhai, 2023). This study also found that 2‐AG levels were significantly increased in the anterior cingulate cortex, caudate putamen, nucleus accumbens, and piriform cortex of mice that underwent restraint stress compared to non‐stressed mice, which agrees with knowledge about the role of 2‐AG following acute stress (Evanson, 2010; Hill & Tasker, 2012; Hill, 2011; Ney, 2018; Wang, 2012). However, this study was only able to detect the potassium adduct of 2‐AG (Zhai, 2023), which is known to interfere with ionisation and efficient quantification of analytes using mass spectrometry (Leite, 2004). Sodium and potassium adducts were quantified using DESI by another group (Islam, 2022), who similarly found that chronic stress significantly increased 2‐AG concentrations in the hindbrain, midbrain, and hypothalamus compared to controls.

In summary, these studies demonstrate exciting possibilities for endocannabinoid spatial quantification using MSI, though also that further method development is critical for the future of this research field.

## 4 CONCLUSION

In conclusion, we conducted a systematic review of articles that have developed new methods for measuring endocannabinoids using targeted LC‐MS/MS in biomatrices over the past 5 years. Our review of the literature shows that it is relatively simple to develop a sensitive assay for quantifying endocannabinoids and related lipids using LC‐MS/MS, even at extremely low abundance with modern equipment. Recent studies have instead shown innovative advances in sample preparation techniques, as well as extension of methods to novel biomatrices and the extended endocannabinoidome. The advancement of these technologies will allow innovative and targeted expansion of MS‐based biomedical research that may improve human health regarding lipids and other biological signalling systems.

## AUTHOR CONTRIBUTIONS

**Khalisa Amir Hamzah**: Conceptualization; data curation; formal analysis; investigation; methodology; resources; software; validation; visualization; writing—original draft; writing—review and editing. **Natalie Turner**: Formal analysis; investigation; writing—review and editing. **David Nichols**: Conceptualization; writing—review and editing. **Luke J. Ney**: Conceptualization; data curation; formal analysis; funding acquisition; investigation; methodology; project administration; supervision; validation; visualization; writing—original draft; writing—review and editing.

## CONFLICT OF INTEREST STATEMENT

The authors declare no conflict of interest.
